# Rare association between spinocerebellar ataxia and amyotrophic lateral sclerosis: a case series

**DOI:** 10.1007/s10072-024-07521-9

**Published:** 2024-04-20

**Authors:** Valerio Ferrari, Matteo Conti, Roberta Bovenzi, Rocco Cerroni, Mariangela Pierantozzi, Nicola B. Mercuri, Alessandro Stefani

**Affiliations:** 1 Parkinson Centre, Department of Systems Medicine, University of Rome “Tor Vergata,”, Rome, Italy; 2 Neurology Unit, Department of Systems Medicine, University of Rome “Tor Vergata,”, Rome, Italy

**Keywords:** Spinocerebellar ataxia, Amyotrophic lateral sclerosis, Motor neuron disease

## Abstract

**Introduction:**

In this work, we describe a new case of association between SCA2 and MND.

**Case Report:**

A 58-year-old man who was diagnosed with spinocerebellar ataxia type 2 presented dysphagia and a significant decline in his ability to walk, with a reduction in autonomy and the need to use a wheelchair. We performed electromyography and electroneurography of the four limbs and of the cranial district and motor-evoked potentials to study upper and lower motor neurons. Referring to the revised El Escorial criteria of 2015, ALS diagnosis was made.

**Discussion:**

Considering different cases described in literature over the years, SCA2 could represent an important risk factor for developing ALS. In particular, the presence of alleles of ATXN2 with 27 and 28 CAG repeats seems to slightly decrease the risk of developing the disease, which would instead be progressively increased by the presence of alleles with 29, 30, 31, 32, and 33 repeats. The exact physiopathological mechanism by which the mutation increases the risk of developing the disease is currently unknown. Transcriptomic studies on mouse models have demonstrated the involvement of several pathways, including the innate immunity regulation by STING and the biosynthesis of fatty acid and cholesterol by SREBP.

**Conclusion:**

CAG repeat expansions in the ATXN2 gene have been associated with variable neurological presentations, which include SCA2, ALS, Parkinsonism, or a combination of them. Further research is needed to understand the relationship between SCA2 and ALS better and explore molecular underlying mechanisms.

## Introduction

Spinocerebellar ataxia type 2 (SCA2) is one of the most common forms of spinocerebellar ataxia (SCA), accounting for approximately 15% of all cases. Several cases of association between SCA and motor neuron disease (MND) have been described over the years, suggesting a possible pathophysiological association between the two disorders [1].

In this paper, we present a case of association between SCA and MND and review the most important features of the previous cases reported in the literature in the most recent years, underlying their similarities and differences.

## Case report

**Fig. 1:**
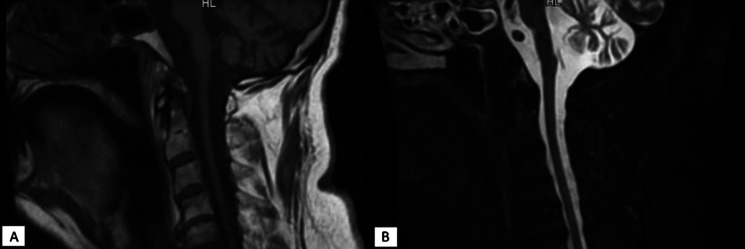
MRI showing marked cerebellar atrophy; **A** T1 sequence; **B** STIR sequence

We describe the case of a 58-year-old Italian man who was diagnosed with SCA2 (38 CAG repeats) at the age of 38 following tests carried out after the onset of progressive postural instability and gait ataxia. Over the years, he developed sensorimotor neuropathy with hypoesthesia and mild weakness in all four limbs. In his family, his father, his uncle, and his first-degree cousin were affected by SCA2, as confirmed by genetic testing. Magnetic resonance imaging (MRI) of the brain with spectroscopy showed cerebellar atrophy and marked reduction of NAA as in chronic gliosis (Fig. 1). Genetic testing confirmed the diagnosis of SCA2.

At the age of 56, following the surgical stabilization of a post-traumatic malleolar fracture, he presented a significant decline in his standing ability and a reduction in walking autonomy, resulting in the need to use a wheelchair. He also reported the presence of cramp-like pain in the four limbs, prevalent in the evening hours. A worsening of the weakness affecting the upper limbs was also reported.

He was admitted to the Neurology Department at I.R.C.S.S. Neuromed. Neurological examination showed difficulties in maintaining an upright position, with the possibility of only taking a few steps with double support. Romberg’s test was positive. Marked limitation of extrinsic eye movements was observed in all directions, with slowed saccadic movements. Speech was severely dysarthric. He presented severe limb ataxia and cerebellar dysmetria in both the upper and lower limbs. The sensibility exam showed superficial tactile hypoesthesia and hypopallesthesia in all four limbs. Deep tendon reflexes were absent. Muscle tone was reduced in all four limbs, and both brachial biceps were hypotrophic. Additionally, fasciculations were observable in all four limbs.

Blood tests showed increased creatine phosphokinase (220 U/L, nv < 177 U/L). TSH was normal. CSF neurodegeneration panel (Abeta 1–42, total tau, p-tau) was not performed since the patient did not give consent for the lumbar puncture.

The otolaryngological evaluation highlighted the presence of severe dysphagia.

Electromyography and electroneurography examination highlighted the presence of severe sensorimotor axonal polyneuropathy and abundant denervation activity in progress in all the muscles investigated in the upper limbs (C5-T1), and signs of chronic neurogenic pain, without denervation in the lower limbs. Motor-evoked potentials showed a reduction in the amplitude and a slight increase in the latency of the cortical stimulus responses with chronodispersed morphology in the left upper and lower limbs and non-reproducible cortical stimulus responses in the right upper limb.

Cognitive impairment manifestations, including psychomotor agitation, incongruous speech and behavior, and sundowning, were also present, yet formal cognitive assessment was not possible due to insufficient cooperation.

According to this data and referring to the revised El Escorial criteria, a diagnosis of probable amyotrophic lateral sclerosis (ALS) was made [2].

Genetic testing for the most common ALS mutation resulted negative (C9orf72, SOD1, TARDBP, FUS).

Subsequent 6-month outpatient follow-up revealed a rapid and progressive decline in neurological and clinical status, marked by complete loss of ambulation, worsening dysphagia and dysarthria, and increased severity and distribution of muscular hypotrophy.

## Case series

An in-depth literature search was performed. Seven papers were selected [3–9], describing a total of eight patients with clinical association of SCA and MND. Table 1 reports all 11 patients’ main demographic and clinical features, including our case report. Cases were numbered from one to 11 according to the year of publication.

**Table 1:** Demographic and clinical features of the cases reported in literature

Author	*Ohara*	*Infante*	*Furtado*	*Nanetti*	*Braga*	*Jaques*	*Nezhad*	*Ferrari*
Report year	1999	2004	2004	2009	2011	2020	2020	2023
Case number	Case 1	Case 2	Case 3	Case 4	Case 5	Case 6	Case 7	Case 8
Patient								
Sex	F	F	M	F	M	M	F	M
Age at SCA onset	57	61	70 s	50	45	58	67	38
Age at MND onset	58	61	70 s	66	46	65	67	58
Age of death	64	64	-	68	-	-	-	-
First SCA symptoms and signs	Dysarthria, gait instability	Dysarthria, gait instability, dysphagia	Dysarthria	Gait instability	Gait instability	Ataxia	Gait instability	Gait instability
First MND symptoms and signs	Atrophy and weakness of the left leg	Generalized weakness	Weakness and atrophy of the right hand	Atrophy of the I interosseus of the left hand	Muscle cramps	Generalized weakness and fasciculations	Weakness of the right hip flessors	Legs weakness
Family history	+	+	+	-	+	+	-	+
DNA analysis	SCA 6	SCA 2	SCA 2	SCA 2	SCA 2	SCA 3	SCA 2	SCA 2
CAG repeats	14/24	35/22	33/22	39/22	40/22	66/ND	32/22	38/22
Motor neuron disease								
Bulbar signs	-	+	-	-	+	-	+	+
Upper motor neuron signs	+	-	+	+	+	-	-	+
Lower motor neuron signs	+	+	+	+	+	+	+	+
Cerebellar ataxia								
Limb ataxia	+	+	-	+	+	+	+	+
Truncal ataxia	-	-	-	-	-	-	-	+
Extraocular movement								
External ophtalmoplegia	-	-	-	+	-	-	-	+
Saccadic pursuit	-	+	+	+	+	+	-	+
Nystagmus	+	-	-	-	-	-	+	+
Parkinsonism								
Bradykinesia	-	+	+	+	ND	-	-	-
Rigidity	-	+	+	-	ND	-	-	-
Tremor	-	-	+	-	ND	-	-	-

*ND*, not determined; *SCA*, spinocerebellar ataxia

The male/female incidence ratio was 1.3/1. The mean age of onset of SCA was 56.4 ± 11.1 years. The mean age of onset of MND is 62.0 ± 8.0 years. To note, the age of case 4 was estimated as 75 years, being reported as “in his seventies” by the author. The mean time difference between the onset of SCA and MND is 5.6 ± 7.5 years, with SCA symptoms developing before or simultaneously but never after MND symptoms in all the cases reported. Regarding the geographical origin of the examined subjects, one case is of Japanese origin, two cases of Italian origin, one Brazilian case of Italian origin, and one Caucasian case with an unknown country of origin. No data regarding the origin of the remaining six cases were available.

### Spinocerebellar ataxia

All cases described are carriers of a mutation detected with genetic testing. Notably, six out of eight patients (75%) were diagnosed with SCA2, one (12.5%) with SCA3, and one (12.5%) with SCA6. SCA2 patients presented with a pathological number of repetitions between 32 and 40. These findings are consistent with previous studies, showing that ataxic phenotype occurs when the repeat is larger than 34 CAG, while repeats between 32 and 34 fall in the gray zone for penetrance and can be associated with a pathological phenotype [10]. Furthermore, alleles with 27–31 CAG were somatically unstable [11].

Out of the reported cases, case 4 is the only one that did not present with limb or truncal ataxia.

Extrinsic ocular motility disorders were reported in all the cases described: the most frequent are represented by pathological saccadic pursuit (63.6%), followed by external ophthalmoplegia (36.4%) and nystagmus (27.3%).

### Motor neuron disease

The involvement of the lower motor neuron was clinically or instrumentally documented in all the cases described. The upper motor neuron was involved in eight of the 11 cases (72.7%). Seven cases also presented clinical involvement of the bulbar district (63.6%). Cases 1, 2, and 4 died of MND-related complications. To our knowledge, all the other cases were alive at the time of their report.

### Parkinsonism

Five patients developed Parkinsonism during the course of the disease (45.5%). Case 2 presented with anterocollis, rigidity of the four limbs, bradykinesia, and abolition of postural reflexes about 6 months after the onset of SCA. Case 3 is the only one who developed tremor, described as asymmetric. One of the three cases of Parkinsonism is reported as levodopa responsive (case 2).

## Discussion

The possible correlation between ATXN2 gene mutations and MND was first hypothesized by Elden et al. [12]. In his study, a cohort of ALS patients presented a double incidence of ataxin-2 with polyglutamine in the upper normal range (> 24 repeats) compared to the control group. It appears that a number of repeats of 31, 32, or 33 could represent an important risk factor for the development of ALS [13], while a number of repeats of 27 or 28 could represent a protective factor [14]. It is interesting to note that, among the cases described in our series, only case 3 and case 7 carried a number of repetitions considered a risk factor for the development of MND.

Corrado et al. found a significant increase in the mean length of CAG repeats in the ATXN2 gene in a group of ALS patients compared to the group of healthy controls. No subject in the control group showed a pathological increase in CAG repeats while four subjects in the ALS group (1.7%) carried an ATXN2 gene with a pathological number of repeats (32, 33, 33, and 37 repeats), each interrupted by at least one CAA triplet [15]. The possible role of such interruptions in determining the disease phenotype (ataxic syndrome/Parkinsonism/MND), together with the length of the CAG repeats, has recently been further investigated by several studies [16].

Moreover, previous studies demonstrated that an intermediate-length CAG number of repeats (31–32) of ATXN2 seems to determine a specific disease phenotype characterized by a more common spinal onset, a more rapid clinical progression and spreading of symptoms, a higher risk of developing cognitive impairment, and a 1-year shorter survival compared to ALS ATXN2 patients [17] [18].

The exact physiopathological mechanism leading to the increased risk of developing MND is currently unknown. The reduction of ATXN2 gene expression in TDP-43 ALS mouse models has been shown to prolong survival by reducing the level of the TDP-43 aggregates [19]. Transcriptomic studies on mouse models have demonstrated the involvement of several pathways, including the innate immunity regulation by STING and the biosynthesis of fatty acid and cholesterol by SREBP [20].

Therapeutic targets capable of decreasing ATXN2 expression levels are currently being researched. The use of antisense nucleotides targeting the ataxin-2 has recently entered a clinical trial in humans [21].

Not much is known about the correlation between other forms of SCA and MND. Three potential pathogenic mechanisms were recently proposed regarding polyglutamine expansion disease (like SCA1, SCA2, SCA3, SCA6, SCA7, and SCA17): the alteration of the protein homeostasis, a dysfunction of the mechanisms of DNA repair, and a dysfunction of the glial system [22]. Under certain circumstances, these alterations could also have repercussions on the motor neuron system, leading to the development of an MND phenotype.

## Conclusion

MND, together with Parkinsonism, represents a rare phenotype of presentation of different types of SCA. Signs of involvement of upper and lower motor neurons should carefully be researched in SCA patient as they could represent a negative prognostic marker in the evolution of the disease. Multidisciplinary management is suggested to prevent all the possible related complications and ensure a better quality of life for the patient.

## Data Availability

Data not published within this article will be made available by request from any qualified investigator.

## References

[1] Antenora A, Rinaldi C, Roca A et al (2017) The multiple faces of spinocerebellar ataxia type 2. Ann Clin Transl Neurol 4:687–695 28904990 10.1002/acn3.437 PMC5590519

[2] Brooks BR, Miller RG, Swash M, Munsat TL (2000) El Escorial revisited: Revised criteria for the diagnosis of amyotrophic lateral sclerosis. Amyotrophic Lateral Sclerosis 1. 10.1080/146608200300079536 10.1080/146608200300079536 11464847

[3] Ohara S, Tsuyuzaki J, Hayashi R et al (2000) Motor neuron loss in a patient with spinocerebellar ataxia type 6: chance co-occurrence or causally related? J Neurol 247:386–388. 10.1007/s004150050608 10896273 10.1007/s004150050608

[4] Ghahremani Nezhad H, Franklin JP, Alix JJP et al (2021) Simultaneous ALS and SCA2 associated with an intermediate-length ATXN2 CAG-repeat expansion. Amyotroph Lateral Scler Frontotemporal Degener 22:579–582. 10.1080/21678421.2020.1853172 33284045 10.1080/21678421.2020.1853172

[5] Jaques CS, Pedroso JL, da Rocha AJ et al (2021) Spinocerebellar ataxia type 3 presenting simultaneously with motor neuron disease and cerebellar ataxia. Arq Neuropsiquiatr 79:851–852 34133492 10.1590/0004-282x-anp-2020-0189

[6] Furtado S, Payami H, Lockhart PJ et al (2004) Profile of families with parkinsonism-predominant spinocerebellar ataxia type 2 (SCA2). Mov Disord 19:622–629 15197699 10.1002/mds.20074

[7] Dayes LA, Gardiner N (2005) The neurological implications of fibromuscular dysplasia. Mt Sinai J Med 72(6) 16358170

[8] Nanetti L, Fancellu R, Tomasello C et al (2009) Rare association of motor neuron disease and spinocerebellar ataxia type 2 (SCA2): a new case and review of the literature. J Neurol 256:1926–1928. 10.1007/s00415-009-5237-9 19644730 10.1007/s00415-009-5237-9

[9] Sibon I, Burbaud P (2004) Risus sardonicus after thalamic haemorrhage. Mov Disord 19:829–831. 10.1002/mds.10675 15254944 10.1002/mds.10675

[10] Cancel G, Dürr A, Didierjean O et al (1997) Molecular and clinical correlations in spinocerebellar ataxia 2: a study of 32 families. Hum Mol Genet 6(5):709–715. 10.1093/hmg/6.5.709 9158145 10.1093/hmg/6.5.709

[11] Laffita-Mesa JM, Velázquez-Pérez LC, Santos Falcón N et al (2012) Unexpanded and intermediate CAG polymorphisms at the SCA2 locus (ATXN2) in the Cuban population: evidence about the origin of expanded SCA2 alleles. Eur J Med Genet 20(1):41–49. 10.1038/ejhg.2011.154 10.1038/ejhg.2011.154 PMC3234519 21934711

[12] Elden AC, Kim HJ, Hart MP et al (2010) Ataxin-2 intermediate-length polyglutamine expansions are associated with increased risk for ALS. Nature 466:1069–1075. 10.1038/nature09320 20740007 10.1038/nature09320 PMC2965417

[13] Sproviero W, Shatunov A, Stahl D et al (2017) ATXN2 trinucleotide repeat length correlates with risk of ALS. Neurobiol Aging 51:178–e1. 10.1016/j.neurobiolaging.2016.11.010 10.1016/j.neurobiolaging.2016.11.010 PMC5302215 28017481

[14] Neuenschwander AG, Thai KK, Figueroa KP, Pulst SM (2014) Amyotrophic lateral sclerosis risk for spinocerebellar ataxia type 2 ATXN2 CAG repeat alleles: a meta-analysis. JAMA Neurol 71:1529–1534. 10.1001/jamaneurol.2014.2082 25285812 10.1001/jamaneurol.2014.2082 PMC4939089

[15] Corrado L, Mazzini L, Oggioni GD et al (2011) ATXN-2 CAG repeat expansions are interrupted in ALS patients. Hum Genet 130:575–580. 10.1007/s00439-011-1000-2 21537950 10.1007/s00439-011-1000-2

[16] Kim J-M, Hong S, Gyoung et al (2007) Importance of low-range CAG expansion and CAA interruption in SCA2 Parkinsonism. Arch Neurol 64(10):1510–1518. 10.1001/archneur.64.10.1510 10.1001/archneur.64.10.1510 17923635

[17] Chiò A, Calvo A, Moglia C et al (2015) ATXN2 polyQ intermediate repeats are a modifier of ALS survival. Neurology 84(3):251–258. 10.1212/WNL.0000000000001159 25527265 10.1212/WNL.0000000000001159

[18] Chio A, Moglia C, Canosa A et al (2022) Exploring the phenotype of Italian patients with ALS with intermediate ATXN2 polyQ repeats. J Neurol Neurosurg Psychiatry 93(11):1216–1220. 10.1136/jnnp-2022-329376 36008116 10.1136/jnnp-2022-329376 PMC9606535

[19] Becker LA, Huang B, Bieri G et al (2017) Therapeutic reduction of ataxin-2 extends lifespan and reduces pathology in TDP-43 mice. Nature 544:367–371. 10.1038/nature22038 28405022 10.1038/nature22038 PMC5642042

[20] Scoles DR, Dansithong W, Pflieger LT et al (2020) ALS-associated genes in SCA2 mouse spinal cord transcriptomes. Hum Mol Genet 29:1658–1672. 10.1093/hmg/ddaa072 32307524 10.1093/hmg/ddaa072 PMC7322574

[21] Kim G, Nakayama L, Blum JA et al (2022) Genome-wide CRISPR screen reveals v-ATPase as a drug target to lower levels of ALS protein ataxin-2. Cell Reports 41(4). 10.1016/j.celrep.2022.111508 10.1016/j.celrep.2022.111508 PMC9664452 36288714

[22] McLoughlin HS, Moore LR, Paulson HL (2020) Pathogenesis of SCA3 and implications for other polyglutamine diseases. Neurobiol Dis 134:104635. 10.1016/j.nbd.2019.104635 10.1016/j.nbd.2019.104635 PMC6980715 31669734

